# Reduced protein levels in latex gloves may play an alternative approach to lowering latex sensitization risks among health workers; a cross-sectional analytical study

**DOI:** 10.1186/s12995-024-00420-x

**Published:** 2024-06-03

**Authors:** Chatpong Ngamchokwathana, Naesinee Chaiear, Jitladda Sakdapipanich, Sumalai Dechyotin, Somsamai Sripramai, Prapassorn Khajornpipat

**Affiliations:** 1 Division of Community Family and Occupational Medicine, Faculty of Medicine, Khon Kaen University, Khon Kaen, Thailand; 2 Rubber Research Group, Department of Chemistry, Faculty of Science, Mahidol University, Nakhon Pathom, Thailand; 3 Clinical Laboratory Section, Faculty of Medicine, Srinagarind Hospital, Khon Kaen University, Khon Kaen, Thailand; 4 Nursing Department, Faculty of Medicine, Srinagarind Hospital, Khon Kaen University, Khon Kaen, Thailand; 5 Nursing Department, Queen Sirikit Heart Center of the Northeast, Faculty of Medicine, Khon Kaen University, Khon Kaen, Thailand

**Keywords:** Glove allergy, Latex sensitization, Latex allergy, Nursing staff, Latex gloves

## Abstract

**Background:**

Latex gloves are essential for protecting healthcare workers from biological hazards but pose a risk of latex allergy, particularly due to powdered, protein, and allergen content. Recent advancements in latex glove manufacturing have led to reduced levels of extractable proteins, a known factor triggering allergenic reaction. This study aimed to compare latex sensitization between nursing staff using low-protein and high-protein latex gloves at a tertiary university hospital in Thailand.

**Methods:**

A cross-sectional analytical study categorized participants into two groups based on glove exposure: the low extractable protein group (only exposed to non-powdered latex gloves with extractable protein levels below 50 µg/g) and the high extractable protein group (exposed to powdered latex gloves with levels above 50 µg/g). The sample size comprised 163 individuals in the low protein group and 318 in the high protein group (1:2). Latex allergy symptoms and sensitization were assessed using a self-administered questionnaire and latex-specific IgE measurement (ImmunoCAP), respectively. Data analysis involved descriptive and inferential statistics, including odds ratios and 95%CI.

**Results:**

Demographic data was mostly similar in both exposure groups except for age. No significant differences in latex sensitization between the low and high protein groups were found via latex-specific IgE measurement (crude OR 1.90, 95%CI: 0.5, 7.18), potentially attributed to lower extractable protein levels in powdered latex gloves compared to previous studies. In contrast, the low protein group exhibited significantly fewer current latex allergy symptoms in both bivariate (crude OR 0.24, 95%CI: 0.06, 0.74) and multiple variable analysis (adjusted OR 0.18, 95%CI: 0.04, 0.86). Moreover, there was a significant reduction in latex allergy symptoms among the low protein group, decreasing from 9.8% who reported experiencing symptoms (when powdered latex gloves were used) to 1.2% who still reported current symptoms (OR 0.11, 95%CI: 0.02, 0.44).

**Conclusions:**

This study underscores the importance of using non-powdered and low-protein latex gloves to reduce latex allergy symptoms while emphasizing the need for further investigation into the relationship between extractable protein levels in addition to the attempt of the major allergen removal and latex sensitization amid evolving glove manufacturing practices.

## Introduction

Latex gloves are widely used as personal protective equipment in healthcare settings due to their favorable properties, including protection against biological hazards, elasticity, and dexterity [1]. Since the late 1980s, the use of latex gloves in healthcare has increased, leading to a continuous rise in latex sensitization and allergy among health workers, becoming a significant occupational health concern [2]. In 2014, the prevalence of latex sensitization and allergy among health workers was estimated to be 12.4% and 9.7%, respectively [3].

Latex allergen exposure can occur through direct skin contact or inhaling latex aero-allergen generated from powdered latex gloves. Implementation of a non-powdered latex glove policy has been identified as an effective measure to decrease the incidence of latex sensitization and allergy [4, 5]. The rationale behind this lies in the aerosolization of powder from the powdered latex gloves, which creates an environment for latex allergens to become airborne and subsequently inhaled [6]. In contrast, non-powdered gloves do not generate this aero-allergen, leading to a decrease in the risk of inhalation and subsequent sensitization [6, 7].

Moreover, extractable protein levels are believed to be a significant contributor to latex sensitization, as they have a reasonable correlation with latex allergen levels [8]. Consequently, many glove standards mandate minimizing extractable protein levels to reduce the risk of latex sensitization. For example, the European standard EN 455-3 stipulates that latex gloves should have leachable protein levels as low as reasonably practicable (less than 50 µg/g) to minimize sensitization and allergy risks [9, 10].

Although, many developed countries have successfully implemented policies on the replacement of powdered latex gloves with non-powdered latex or synthetic rubber gloves through laws and regulations, which effectively reduced latex sensitization and allergy cases [9, 11], this replacement is not fully implemented in developing countries due to economic constraints. For instance, in Thailand, while the Ministry of Public Health has banned the use of sterile powdered latex gloves, disposable powdered latex gloves remain in use by health workers [12]. This has led to persistent problems of latex sensitization and allergy among health workers in developing countries.

While previous studies have shown the effectiveness of policies banning powdered latex gloves in reducing latex sensitization and allergy, there remains a gap in understanding the relationship between extractable protein levels and latex sensitization and allergy. To address this gap, this study aims to investigate latex sensitization among nursing staff in two distinct exposure groups (low vs. high): those regularly using non-powdered latex gloves with lower extractable protein levels (less than 50 µg/g), and those using powdered latex gloves with higher extractable protein levels (more than 50 µg/g). Understanding this association is crucial, as extractable protein levels play a significant role in latex allergy issues, influencing policies aimed at promoting the use of latex gloves with lower extractable protein levels. This not only contributes to a safer workplace for health workers but also leads to cost savings within the healthcare sector, especially in developing countries.

## Materials and methods

This study was a cross-sectional analytical study conducted in two healthcare facilities in Northeastern Thailand, at Srinagarind Hospital and Queen Sirikit Heart Center of the Northeast, Khon Kaen University, Khon Kaen province, Thailand. Data collection and participant recruitment occurred within the timeframe of January to April 2022.

### Study population

The sample size for this study was determined based on previous research by M. Joan Saary et al. [13], which identified that individuals exposed to powdered latex gloves had a prevalence of latex sensitization of 10%, while those exposed to non-powdered latex gloves (that generally contained lower extractable protein) had a prevalence of 3%. The sample size was calculated using Winpepi version 11.65, with a 1:2 ratio between the low protein latex gloves exposure and high protein latex gloves exposure groups, a significance level of 5%, and a power of 80%. The calculated sample size for the study was 154 in the low protein group and 308 in the high protein group. However, to account for potential losses, the sample size was increased by 20% to 193 participants in the low protein group and 386 participants in the high protein group.

The study participants were nursing staff who had worked at one of those two hospitals for at least six months and were divided into two groups based on their exposure to latex gloves with different extractable protein levels. Following the EN455-3 recommendation, which suggests extractable protein should be ≤ 50 µg/g for non-powdered latex gloves [10], A threshold of 50 µg/g was used to differentiate between high and low protein levels in latex glove exposure in our study. The modified Lowry method was used to confirm the extractable protein levels in latex gloves currently used in our setting. A total of four pairs of each glove type—including nonsterile powdered latex gloves, nonsterile non-powdered latex gloves, sterile powdered latex gloves, and sterile non-powdered latex gloves—were collected from various departments. Following the collection, the extractable protein levels were assessed in accordance with the European standard (EN 455-3) using the modified Lowry method. This analysis was conducted by the Department of Science Service, located in Bangkok, Thailand. The “low protein group” comprised 212 operating theatre nurses who exclusively used non-powdered or synthetic rubber gloves, using a minimum of three pairs per day. The non-powdered latex gloves in this group exhibited protein levels ranging from < 9.9 to 36.7 µg/g. The “high protein group” consisted of 1,455 staff spread across 54 units, including inpatient, ICU, and emergency departments. Nursing staff in this group were typically required to use powdered latex gloves with protein levels ranging from 53.0 to 56.9 µg/g with a minimum of three pairs per day, although they were also allowed exposure to gloves with lower protein levels. Cluster sampling was utilized to probabilistically select clusters from the study population. In this process, nursing staff from 15 units were chosen as the high protein group, comprising a total of 412 nursing staff members.

### Research tools

The research tools used in this study included self-administered questionnaires and blood samples for serum latex-specific IgE level analysis. The questionnaires were used to evaluate latex allergy symptoms, including cutaneous and non-cutaneous symptoms, as well as demographic data, underlying atopic diseases, a history of fruit allergy (latex-fruit syndrome), glove exposure characteristics, and other latex product exposure. The questionnaire component was derived and adapted from previous studies conducted by Chaiear et al. [14] and Boonchai et al. [15]. It was reviewed by three experts in dermatology, immunology, and occupational medicine to ensure its validity. The questionnaire’s item-objective congruence was found to be 0.92. Additionally, its test-retest reliability was determined by having 30 nursing staff complete the questionnaire twice with a two-week interval, resulting in a stability coefficient of 0.996. Participants who reported symptoms of latex allergy, including both cutaneous (e.g., urticaria, erythema rash, or itching) and non-cutaneous symptoms (e.g., runny nose, dyspnea, wheezing, or conjunctival injection) after being exposed to latex gloves within 24 h were classified as having latex allergy symptoms.

Blood samples were obtained and analyzed to determine serum-specific IgE levels at the clinical laboratory section of Srinagarind Hospital in Khon Kaen. The ImmunoCAP and the Phadia 250 analyzer were used. This laboratory technique measures the level of IgE specific to natural latex allergens through a fluoroenzyme immunoassay with a sensitivity of 76.3% and a specificity of 96.7%. Latex sensitization was defined as serum latex-specific IgE levels equal to or exceeding 0.35 kAU/L [16].

### Data collection

All participants mentioned in the study population section were explained the research process, benefits, and risks of participating in this study. Informed consent was obtained from each participant before administering the questionnaire and collecting blood samples. The self-administered questionnaire, in its online format, was completed by the participants. Following the questionnaire, blood samples were taken on the same working day. These samples were promptly sent to the laboratory unit, where they were centrifuged and stored at a temperature of -20 °C until the time of analysis.

### Statistical analysis

**Fig. 1:**
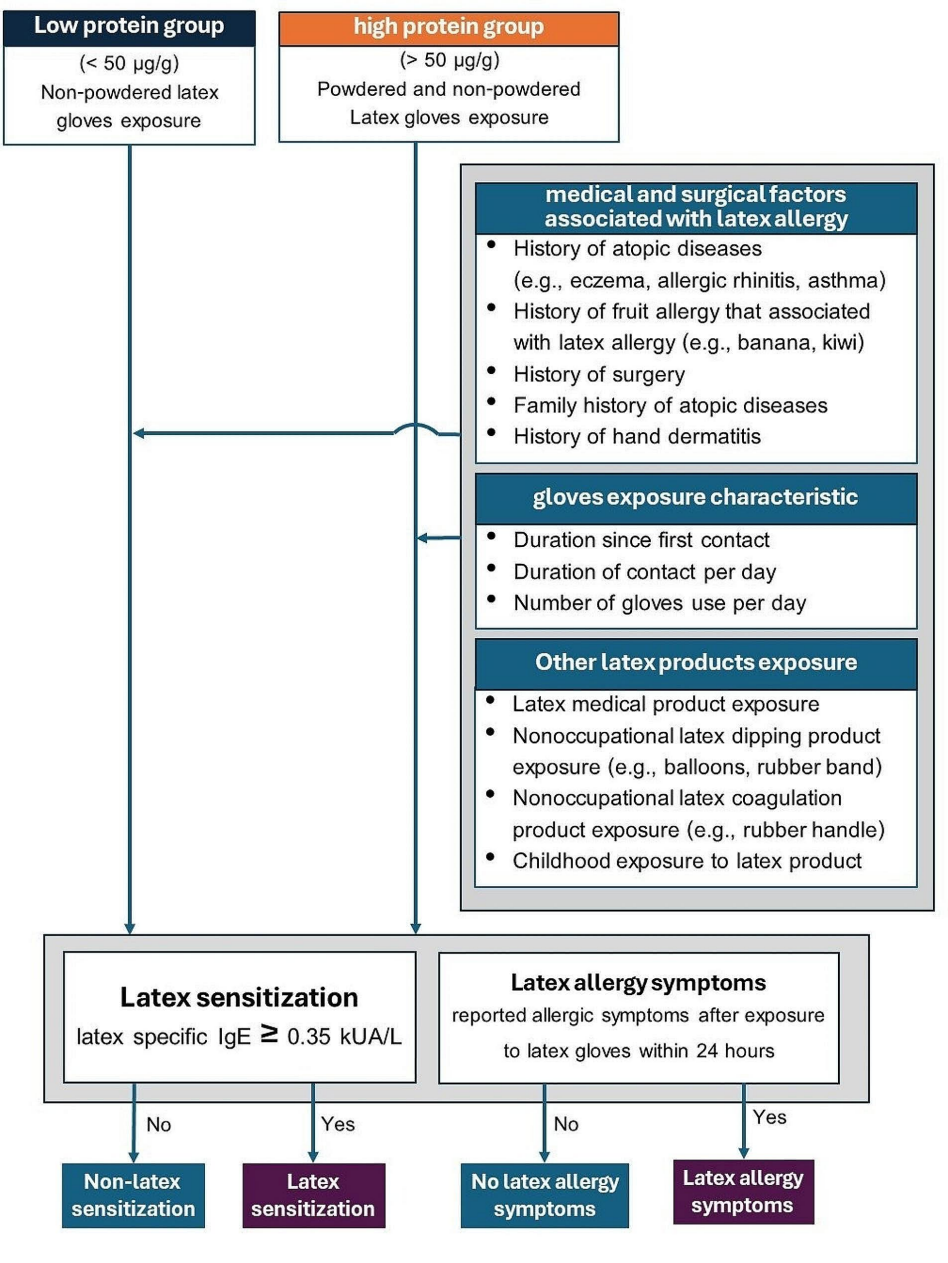
Conceptual framework

The statistical analyses were conducted using IBM SPSS version 28.0 and OpenEpi. Categorical data, such as demographic information, exposure characteristics, and latex allergy symptoms, were presented as proportion and percentage, while continuous data, such as age, employment duration, exposure characteristics, and latex-specific IgE level, were presented as mean and standard deviation (SD) or median and interquartile range (IQR). The relationship between latex sensitization and allergy between the two exposure groups was analyzed using the Chi-square or Fisher’s exact test, and a p-value of less than 0.05 was considered to be statistically significant. Lastly, multiple logistic regression analysis was used. Factors for latex allergy symptoms, including history of atopic diseases, history of fruit allergy, family history of atopic diseases, surgical history, glove exposure characteristics, and other latex product exposure, were analyzed to determine an association. The conceptual framework that describes the details for multiple variable analysis is shown in Fig. 1.

## Results

The response rate of this study was 76.8% (163 out of 212) in the low protein group and 77.2% (318 out of 412) in the high protein group. Of the participants in the low protein group, 58.0% (123 out of 212) agreed to the blood collection for latex-specific IgE, while 56.6% (233 out of 412) of participants in the high protein group did so. Unfortunately, two samples from participants in the high protein group were not suitable for analysis due to inadequate specimens. The median age of participants in the low protein group was 30 years (IQR 25–37) and 32 years (IQR 28–42) in the high protein group. Most of the participants were female and registered nurses. Approximately half of the participants reported underlying atopic diseases. The demographic data and underlying diseases related to latex sensitization are presented in Table 1 and showed no significant differences between the two exposure groups, except for age.

**Table 1:** Demographic data and underlying factors related to latex allergy among two exposure groups

Demographic data	Low protein exposure group *N* = 163 (%, n)	High protein exposure group *N* = 318 (%, n)	*P*-value
**1.** **Age** (years, IQR)	30 (25–37)	32 (28–42)	0.01
**2.** **Sex**			0.33
Female	92.0 (150)	94.3 (300)	
Male	8.0% (13)	5.7% (18)	
**3.** **History of atopic diseases**	50.3% (82)	47.5% (151)	0.56
**4.** **History of fruit allergy**	0.6% (1)	2.5% (8)	0.13
**5.** **History of surgery**	23.3% (38)	29.9% (95)	0.13
**6.** **Family history of atopic diseases**	20.2% (33)	26.1% (83)	0.16
**7.** **History of hand dermatitis**	24.5% (40)	18.9% (60)	0.15

Several exposure factors, such as job characteristics and glove usage patterns (i.e., duration of glove exposure per day and frequency of glove use), exhibited notable differences between the two groups. Additionally, there was a significant disparity in the duration of initial contact with latex gloves between the two exposure groups. Furthermore, variations were observed in the exposure to different types of latex medical products, including tourniquets, elastic bandages, and stethoscopes. However, despite these differences, there were no significant disparities in overall exposure to latex medical products. Further details on the exposure characteristics of gloves and other latex products can be found in Table 2.

**Table 2:** Characteristics of latex products and gloves exposure among two exposure groups

Exposure characteristic	Low protein exposure group *N* = 163, % (n)	High protein exposure group *N* = 318, % (n)	*P*-value
**1. Job characteristic**			0.005
1.1 Registered nurse	76.7 (125)	66.4 (211)	
1.2 Nurse assistant	22.7 (37)	29.2 (93)	
1.3 Nursing aid	0.6 (1)	2.8 (9)	
1.4 Hospital housekeeper	0 (0)	1.6 (5)	
**2. Intensity of gloves exposure**			
2.1 Duration since first contact (years, IQR)	8 (3,13.5)	10 (5,15)	0.04
2.2 Duration of contact per day (hours, IQR)	6 (4, 8)	4 (2,4)	< 0.001
2.3 Frequency of contact per day (pairs, IQR)	7 (5, 10)	10 (6,15)	< 0.001
**3. Occupational latex medical product exposure and type**	68.7 (112)	73.9 (235)	0.22
3.1 Tourniquet	34.4% (56)	59.7% (190)	< 0.001
3.2 Elastic bandage	58.9% (96)	36.2% (115)	< 0.001
3.3 Stethoscope	9.2% (15)	46.5% (148)	< 0.001
3.4 other latex medical products	41.1% (67)	39.6% (126)	0.77
**4. Nonoccupational latex exposure by types**			
4.1 Dipping product exposure	40.5% (66)	33.6% (107)	0.14
4.2 Coagulation product exposure	39.9% (65)	44.7% (142)	0.32
**5. Childhood exposure to latex**	81.6% (133)	82.1% (261)	0.90

In terms of latex sensitization, the main outcome of this study, a total of 2.8% (10 out of 354) of all participants across both exposure groups tested positive for latex-specific IgE. When examining the findings in detail, 4.1% (5 out of 123) of participants in the low protein group and 2.2% (5 out of 231) of participants in the high protein group exhibited positive results for latex-specific IgE. Notably, our analysis did not identify any statistically significant differences between these two exposure groups with respect to latex sensitization (OR 1.90, 0.5–7.2). Furthermore, while the geometric mean of latex-specific IgE levels in the low protein group was lower, measuring at 0.0029 kUA/L, compared to the high protein group, which recorded a mean level of 0.0037 kUA/L, these variations were not deemed statistically significant. These results collectively indicate that there was no substantial difference in latex sensitization prevalence or mean latex-specific IgE levels between two exposure groups. Additionally, it’s noteworthy that out of the ten nursing staff members who tested positive for latex sensitization, only two of them reported experiencing current latex allergy symptoms. Importantly, both of these individuals belonged to the high protein group.

Based on the findings obtained from a self-administered questionnaire, our study revealed that 1.2% of participants in the low protein group reported experiencing current latex allergy symptoms, compared to 7.2% of participants in the high protein group. This significant difference in current latex allergy symptoms was quantified using an OR of 0.16 (95%CI: 0.03, 0.59), indicating a substantially lower odds ratio of experiencing such symptoms in the low protein group in comparison to the high protein group. Detailed findings on latex allergy symptoms are presented in Table 3. Additionally, when focusing solely on participants in the low protein group, a significant reduction in latex allergy symptoms was observed when comparing previous and current latex allergy symptoms (OR 0.11; 95% CI: 0.02, 0.44). While 9.8% of participants in the low protein group reported experiencing latex allergy symptoms, which may occur in the past when powdered latex gloves have been used in the operating theatre, only 1.2% of participants reported current symptoms, after the ban on powdered latex gloves. This finding suggests a potential protective effect against latex allergy symptoms after removing powdered latex gloves from the environment.

**Table 3:** Latex allergy symptoms categorized by exposure groups

Latex allergy symptoms	Low protein exposure group *N* = 163, n (%)	High protein exposure group *N* = 318, n (%)	OR (95%CI)	*P*-value
**Experience of latex allergy symptoms**	16 (9.8)	31 (9.7)	1.01 (0.52, 1.89)	0.97
- Cutaneous symptoms	11 (6.8)	29 (9.1)	0.72 (0.34, 1.46)	0.38
- Non-cutaneous symptoms	1 (0.6)	2 (0.6)	0.98 (0.03, 12.9)	0.98
**Current latex allergy symptoms**	2 (1.2)	23 (7.2)	0.16 (0.03, 0.59)	0.002
- Cutaneous symptoms	2 (1.2)	21 (6.6)	0.18 (0.03, 0.66)	0.005
- Non-cutaneous symptoms	0 (0)	2 (0.6)	1.29 (0.04, 19.92)	0.84

Furthermore, our research identified two significant contributing factors to the occurrence of latex allergy symptoms: a history of hand dermatitis with an OR 2.70 (95%CI: 1.14, 6.24) and the frequency of glove contact per day (p-value = 0.02). Additionally, our result found that the frequency of glove contact for more than eight pairs per day increases the risk of developing latex allergy symptoms with an OR 3.02 (95%CI: 1.22, 8.41). To establish a more robust understanding of these associations, multiple logistic regression analysis was employed, which confirmed the earlier observations. Even after controlling for associated medical/surgical history, intensity of glove exposure, and exposure to other latex products, our study still demonstrated significantly lower current latex allergy symptoms in the low protein compared to the high protein group, with an adjusted OR of 0.18 (95%CI: 0.04, 0.86). More detailed findings are presented in Table 4.

**Table 4:** Summary of the association between factors and current latex allergy symptoms

Associated factors	Crude OR (95%CI)	Adjusted OR (95%CI)	*P*-value
**Exposure intensity**			
Exposure group (low protein, high protein)	0.24 (0.06, 0.74)	0.18 (0.04, 0.86)	0.03
**Medical and surgical factors**			
- History of atopic diseases (no, yes)	0.98 (0.43, 2.23)	0.91 (0.36, 2.30)	0.84
- History of fruit allergy	2.33 (0.10, 15.42)	3.55 (0.35, 36.1)	0.28
- History of surgery	1.25 (0.50, 2.93)	0.92 (0.33, 2.56)	0.87
- Family history of atopic diseases	1.83 (0.75, 4.25)	1.68 (0.64, 4.37)	0.29
- History of hand dermatitis (no, yes)	2.70 (1.14, 6.24)	2.77 (1.11, 6.96)	0.03
**Glove exposure characteristic**			
- Duration since first contact (year)	N/A	0.99 (0.94, 1.04)	0.63
- Duration of contact per day (hours)	N/A	0.92 (0.33, 2.56)	0.32
- Number of gloves used per day (pairs)	N/A	1.09 (1.01, 1.18)	0.04
**Other latex product exposure**			
- Occupational latex medical product exposure (no, yes)	0.33 (0.14, 0.76)	0.26 (0.10, 0.66)	0.004
- Nonoccupational exposure to dipping products (no, yes)	1.43 (0.62, 3.23)	1.86 (0.69, 5.00)	0.22
- Nonoccupational exposure to coagulation products (no, yes)	1.04 (0.45, 2.37)	0.89 (0.33, 2.37)	0.81
- Childhood exposure to latex (no, yes)	1.17 (0.41, 4.07)	1.38 (0.41, 4.60)	0.60

## Discussion

This cross-sectional analytical study aimed to explore the relationship between latex sensitization and extractable protein levels. The choice of this research design was deemed suitable due to the continuous and natural occurrence of exposure. However, the number of participants undergoing specific IgE measurements was limited, potentially resulting in inadequate statistical power. To address this, the authors attempted to recalculate the sample size, revealing a lower requirement. Notably, a study in Italy reported a significant decrease in positive latex skin prick test cases among healthcare workers following the banning of powdered latex gloves, with prevalence dropping from 5.9 to 0% over eight years [17]. Recalculating our sample size in accordance with these results yielded estimates of 106 participants in the low protein group and 212 in the high protein group, aligning more closely with the actual participant numbers in our study. Moreover, from a biomedical perspective, the lower levels of extractable protein observed in our study compared to previous studies [9, 18, 19] may lead to fewer cases of latex sensitization. Thus, increasing the sample size might not notably change the observed prevalence of latex sensitization in both exposure groups and would likely not distort the overall findings. Additionally, when discussing the research tools in this study, it’s essential to weigh the pros and cons of latex skin prick tests and latex-specific IgE measurement. While latex skin prick tests may offer higher sensitivity and specificity compared to the latter, the lack of standardized reagents in the market poses challenges. This limitation can potentially reduce sensitivity on detecting latex sensitization and increase the risk of adverse reactions [20]. On the other hand, specific IgE measurement, with a demonstrated sensitivity of 76.3% and specificity of 96.7%, provides a reliable and reproducible method for determining latex sensitization [16].

Our study found that while demographic data did not significantly differ between the low and high protein groups, several exposure factors, including job characteristics and glove usage patterns (such as duration of glove exposure per day and frequency of glove use), exhibited notable disparities. This was attributed to the different working characteristics of the participants in the operating theatre and inpatient department. The non-powdered latex gloves exposure group consists of nursing staff primarily stationed in operating theaters, where the nature of their work requires them to wear gloves for extended durations during surgical procedures. In contrast, the powdered latex gloves exposure group comprises nursing staff stationed in inpatient care or intensive care units, where the use of gloves is more frequent but of shorter duration, as they change gloves regularly while attending to patients on a case-by-case basis.

Latex allergy is an IgE-mediated hypersensitivity reaction that arises from exposure to latex allergens. The higher the levels of latex allergen exposure, the higher the chance of developing latex sensitization. However, our study cannot demonstrate the significant differences in latex sensitization between low and high protein groups (4.1% vs. 2.2%). This discrepancy can be attributed to the comparable extractable protein levels in both high (powdered) and low protein (non-powdered) gloves. The lower levels of extractable protein (53.0-56.9 µg/g) observed in powdered latex gloves (classified as high protein gloves in our study) compared to levels reported in previous studies (generally higher than 100 µg/g) [9, 19] may account for the lack of significant differences in latex sensitization cases. In recent years, advancements in latex glove production, such as in-line high-temperature processes and post-washing procedures, have led to a significant decrease in extractable protein levels [9]. A striking contrast can be observed when comparing studies published in 2003 and 2016; the extractable protein levels in latex gloves from Germany in 2003 were as high as 917.38 µg/g, and 68.4% of these gloves exceeded the DGUV recommended limit of 30 µg/g (TRGS 540) [19]. In contrast, a study published in 2016 reported that the extractable protein content in latex gloves in Germany had significantly decreased to 92.3 µg/g, with only 27.8% of all gloves in that study exceeding the DGUV recommended limit [21]. Moreover, our study found lower extractable protein levels in currently used latex gloves (lower limit of detection – 33.6 µg/dm^2^) when compared to the gloves examined in earlier studies in our country (115.1–203.9 µg/dm^2^) [18]. These earlier studies demonstrated a higher prevalence of latex sensitization among health workers (4.7%) compared to our research (2.8%) [18]. This serves as evidence of the technological improvements in glove production, which have likely contributed to the reduction in latex allergen levels and, consequently, the absence of significant differences in latex sensitization observed in our study.

In contrast, our study highlights that nursing staff in the low protein group, who were exposed to non-powdered latex gloves with lower extractable protein, had significantly fewer latex allergy symptoms than those in the high protein group (1.2% vs. 7.2%, OR 0.16, 95%CI: 0.03, 0.59), who were still exposed to powdered latex gloves with higher extractable protein. This difference persisted even after adjusting for various factors, as shown in Table 4 (adjusted OR 0.18, 95% CI: 0.04, 0.86). This phenomenon may be explained by the fact that the use of powdered latex gloves increases the risk of developing latex allergy symptoms [6, 22, 23]. Not only extractable protein in latex gloves but also powdered in latex gloves can increase the risk of latex allergy symptoms. There are various explanations for this. The presence of glove powder can significantly impact skin integrity by inducing roughness and compromising the natural skin barrier, thereby elevating the risk of exposure to latex allergens, and subsequently increasing the likelihood of adverse allergic reactions [24]. This elucidates why individuals in the low protein group, where powder usage was eliminated, reported lower occurrences of current latex allergy symptoms compared to both the high protein group and previous studies. Additionally, powdered latex gloves can produce latex-aeroallergen in the workplace, causing a higher chance of latex-allergen exposure, which leads to a higher prevalence of latex sensitization and latex allergy symptoms [22]. In addition, Baur also confirmed a significant correlation between types of latex gloves (powdered/non-powdered) and airborne latex allergens [25]. Therefore, latex allergy was notably more prevalent in the high protein group where powdered latex gloves were still in use, highlighting the potential role of latex aeroallergen inhalation in triggering latex allergy. Our findings were consistent with many studies. Based on a study conducted in the United States, a noteworthy decrease in symptoms associated with latex glove exposure was observed, with a decline from 42 to 29% following the substitution of powdered latex gloves with non-powdered latex gloves and synthetic rubber gloves [4]. In Canada, the incidence of symptoms also decreased from 20 to 6% after those policies were implemented. Furthermore, a similar outcome was observed in Sweden, the UK, and Germany [6, 26, 27]. However, this result could be interpreted cautiously. Participants in our study may have experienced confusion between the symptoms of latex allergy and those associated with other forms of contact dermatitis. The irritant properties of powdered gloves, along with additives in latex gloves, may contribute to glove-related irritant or allergic contact dermatitis, leading to symptoms like itching and an erythematous rash, which resemble latex allergy symptoms [24, 28, 29]. This could explain the higher reporting rate of symptoms, such as itching and redness, among participants exposed to powdered latex gloves, causing some confusion. Contact dermatitis is the most common occupational skin condition and can affect individuals in various professions, including health workers [30]. A study on latex glove-related skin symptoms showed that nearly all participants (93.2%) who reported glove-related skin symptoms, such as itching, erythema, and dryness, were diagnosed with contact dermatitis, while 2.4% were confirmed to have both contact dermatitis and contact urticaria due to latex, as determined by patch tests and skin prick tests [31]. Similar results were found in a study in India, where 93.2% of participants reporting skin symptoms related to latex glove exposure had contact dermatitis, while only 28.3% were confirmed to have latex allergy, and 21.6% had both contact dermatitis and contact urticaria [32]. These findings provide evidence of potential confusion between the symptoms of latex allergy and contact dermatitis, and in some cases, both conditions may coexist.

While our study provides valuable insights, we recognize limitations. As a cross-sectional analytical study, selection bias, particularly the healthy worker effect, may be present [33]. Nursing staff with latex allergies might have left the workplace due to symptoms from latex glove exposure before data collection. However, nursing entails extensive training and expertise, making it unlikely for professionals to switch fields unnecessarily. Moreover, it’s worth noting that a previous study identified neuropsychological symptoms, such as sleep disturbances or fatigue, as the primary health concerns affecting the turnover rates of nursing staff, not allergies [34]. In addition, data from our faculty’s Occupational Health and Safety unit revealed that few nursing staff experiencing severe latex allergy symptoms, like anaphylaxis, changed careers. Hence, while the healthy worker effect may exist, its impact on our results is minimal. Furthermore, while participants were given autonomy to decide on blood collection, selection bias may still exist. Comparing variables between participants who accepted and refused blood collection revealed higher rates of underlying atopic disease, family history, surgery, and latex allergy symptoms among participants who accepted. This suggests those at higher risk of latex sensitization were more inclined to participate, potentially impacting prevalence rates. Nonetheless, upon comparing demographic data between the low and high protein exposure groups, as depicted in Table 1, there were almost no significant differences in these variables. This suggests that these risk factors may have a minimal effect on our outcome. Lastly, caution is needed when interpreting findings on latex allergy symptoms due to potential misclassification and self-report bias. However, our questionnaire underwent validation and included visuals to mitigate self-report bias.

Unlike clinical settings, the field of occupational health places a strong emphasis on proactive measures aimed at identifying abnormalities and potential health risks before they escalate into full-blown diseases. In the healthcare sector, occupational medicine physicians are pivotal in the early identification of latex sensitization. Their aim is to prevent the onset of latex allergy symptoms and minimize the risk of more severe complications among healthcare workers. Implementing personal protective equipment suitable for workers with latex sensitization is crucial to enhancing the quality of working life for health workers while simultaneously safeguarding their health. Our study suggests that one promising approach to effectively controlling and managing latex sensitization among health workers is to focus on reducing extractable protein levels in latex gloves. This approach presents a viable alternative that has the potential to contribute to the broader goal of minimizing latex-related health risks among this workforce. For instance, in settings where powdered latex gloves are still in use, tailoring the selection of latex gloves based on individual risk factors and symptoms could be a suitable approach. Health workers without risk factors or symptoms of latex allergy, considered a low-risk group, may still find powdered latex gloves with lower extractable protein levels, if available. For those experiencing mild latex allergy symptoms such as itching and/or urticaria, classified as a moderate risk group, providing non-powdered gloves with a lower protein content could be appropriate. Conversely, for individuals at a high risk of severe allergic reactions or those experiencing non-cutaneous symptoms, opting for synthetic rubber gloves and providing non-powdered latex gloves for coworkers could be the most effective strategy. This approach aligns with a previous suggestion from the UK [35] and may be implemented without significantly increasing the cost of gloves in settings where economic constraints are a concern. Additionally, while extractable protein may offer an indirect measurement of latex glove allergenicity compared to other techniques, such as determining latex allergen levels, it is more practical and cost-effective. This makes it feasible to assess protein levels in latex gloves in many settings, especially in developing countries where resources may be limited. By addressing the root cause of latex sensitization and taking proactive steps, such as assessing and replacing gloves with high protein levels with lower ones, we can ensure the well-being and longevity of healthcare professionals in their critical roles within the healthcare industry.

Future studies should delve into the potential factors stemming from the observed extractable protein levels in both the high and low protein groups. Unlike previous studies, where powdered latex gloves typically exhibited higher extractable protein levels, our findings show a closer resemblance in protein levels between the two groups. Thus, further investigation is needed to understand the implications of these protein levels, especially in populations exposed to higher concentrations (> 200 µg/g) in latex gloves. Moreover, comprehensive clinical assessments in future studies can help distinguish true latex allergy from other dermatological reactions, providing a clearer understanding of how glove type influences symptom presentation among healthcare workers.

## Conclusion

In conclusion, this study provides valuable insights into latex sensitization among health workers. While our findings did not demonstrate significant differences between low and high protein groups, an association between the prevalence of latex sensitization and extractable protein levels was observed compared to previous studies. The reduction in extractable protein levels in powdered latex gloves emerged as a crucial factor contributing to the decrease in latex sensitization. Our findings suggest that implementing policies to reduce extractable protein in latex gloves could benefit in reducing latex sensitization cases in healthcare facilities, especially in settings where a total ban on latex gloves is not feasible. Additionally, the elimination of powdered latex gloves has been shown to be a protective measure against the development of latex allergy symptoms. These findings emphasize the importance of adopting non-powdered latex gloves and minimizing extractable protein levels to safeguard the health and well-being of healthcare professionals.

## Data Availability

Additional data of this study are available from the corresponding author upon reasonable request.

## Declarations

### Manuscript preparation

In the process of preparing this article, ChatGPT-3.5, a Large Language Model Al tool, was used to improve and refine the language used in this article. However, it is crucial to emphasize that all the content, ideas, and concepts presented in this study are entirely the original work is the author’s own idea, with no instances of potential plagiarism.
